# Myoinositol CEST signal in animals with increased Iba-1 levels in response to an inflammatory challenge—Preliminary findings

**DOI:** 10.1371/journal.pone.0212002

**Published:** 2019-02-21

**Authors:** Maria Yanez Lopez, Marie-Christine Pardon, Kerstin Baiker, Malcolm Prior, Ding Yuchun, Alessandra Agostini, Li Bai, Dorothee P. Auer, Henryk M. Faas

**Affiliations:** 1 Sir Peter Mansfield Imaging Centre, School of Medicine, The University of Nottingham, Nottingham, United Kingdom; 2 School of Life Sciences, University of Nottingham, Nottingham, United Kingdom; 3 School of Veterinary Sciences, University of Nottingham, Nottingham, United Kingdom; 4 Medical Imaging Unit, University of Nottingham, Nottingham, United Kingdom; 5 School of Computer Sciences, University of Nottingham, Nottingham, United Kingdom; Johns Hopkins University, UNITED STATES

## Abstract

Neuroinflammation plays an important role in the pathogenesis of a range of brain disorders. Non-invasive imaging of neuroinflammation is critical to help improve our understanding of the underlying disease mechanisms, monitor therapies and guide drug development. Generally, MRI lacks specificity to molecular imaging biomarkers, but molecular MR imaging based on chemical exchange saturation transfer (CEST) can potentially detect changes of myoinositol, a putative glial marker that may index neuroinflammation. In this pilot study we aimed to investigate, through validation with immunohistochemistry and *in vivo* magnetic resonance spectroscopy (MRS), whether CEST imaging can reflect the microglial response to a mild inflammatory challenge with lipopolysaccharide (LPS), in the APPSwe/ PS1 mouse model of Alzheimer’s disease and wild type controls. The response to the immune challenge was variable and did not align with genotype. Animals with a strong response to LPS (Iba1+, n = 6) showed an increase in CEST contrast compared with those who did not (Iba1-, n = 6). Changes of myoinositol levels after LPS were not significant. We discuss the difficulties of this mild inflammatory model, the role of myoinositol as a glial biomarker, and the technical challenges of CEST imaging at 0.6ppm.

## Introduction

Neuroinflammation is a potential factor in a range of neurodegenerative diseases, including stroke, Parkinson’s and Alzheimer’s disease (AD) [1–3]. Microglia, key mediators of the CNS response to an inflammatory stimulus [4], react to pathological insults by undergoing a transformation towards an activated state, changing morphology, accumulating at the site of the inflammatory insult, and triggering the cascade of molecular events that characterizes neuroinflammation. In the healthy brain, this process is finely regulated, but in pre-existing neurodegenerative conditions the microglial response can exacerbate disease progression [5, 6]. Therefore, glial activation and neuroinflammation are potentially key modifiable mechanisms for different neurodegenerative disorders.

Non-invasive imaging biomarkers are important tools for the development of potential new treatments and for monitoring therapeutic effects. Radiolabeled ligands for positron emission tomography (PET) based on the upregulation of the translocator protein 18kDa (TSPO) have been shown to allow detection and monitoring of neuroinflammation [7, 8]. Due to its versatility, absence of ionizing radiation and routine clinical use, MRI would be a particularly attractive modality to complement PET to image neuroinflammation non-invasively. To date, however, MR imaging is generally not sensitive enough to monitor molecular markers of neuroinflammation.

MR spectroscopy on the other hand allows to measure a range of metabolites that have been linked to glial activation and neuroinflammation, including creatine, choline, glutamate, and myoinositol (mI) [9–11]. But the spatial resolution of MR spectroscopy is poor, and an MR imaging approach that retains spatially resolved molecular information indicative of neuroinflammation would be highly valuable. One way of overcoming the limitation of MR spectroscopy while to a degree retaining its specificity is to use chemical exchange saturation transfer (CEST), where proton exchange between low-concentration molecules—below the detection threshold of standard MRI—containing hydroxyl (-OH), amine (-NH_2_), or amide groups with the abundant water protons amplifies the signal of a target molecule [12]. Several studies have explored the possibility of imaging a neuroinflammatory response with CEST MRI, focusing on strong inflammatory stimuli or inflammation in late stages of disease. A reduction in CEST contrast at 3 ppm (attributed predominantly to glutamate changes and hence named GluCEST) was seen to correlate with a decrease in glutamate levels in 18–20 month old animal models of AD [13]. Similar changes in the GluCEST signal have been demonstrated in a model of astrocyte activation (overexpression of the cytokine ciliary neurotrophic factor, CNTF, a strong activator of astrocytes) [14]. A reduced GluCEST signal has also been reported in a 20-month old mouse model of tauopathy [15], in a 12-month old mouse model of Huntington disease [16] and in patients on the psychosis spectrum [17], while increased gluCEST signal was observed in a mouse model of dopamine deficiency [18].

Myoinositol is primarily found in glial cells [9] and has therefore been proposed as a marker for microglial activation [19]. An increase in myoinositol levels in MR spectroscopy studies could be explained by a microglial proliferation observed in the neuroinflammatory response [20–22]. Myoinositol is suitable for detection with CEST, because its six hydroxyl protons exchange with bulk water at a rate of k = 600 s^−1^, and a frequency shift Δω < 1 ppm from bulk water, placing it in the slow to intermediate NMR exchange regime. This was first demonstrated by Haris et al. [23], who later also described an increase in the CEST signal at 0.6 ppm in an 18–20 month old AD mouse model [13]. Higher concentrations of myoinositol in Alzheimer’s disease have also been demonstrated in the very early stages of disease progression [24]. An increase in myoinositol levels has been consistently observed in the posterior cingulate, hippocampus and anterior cingulate gyrus in mild cognitive impairment in the early stage of dementia [22]. Together these studies suggest that myoinositol levels assessed by MR spectroscopy may index neuroinflammation and microglial activation. However, recent studies question the relationship between myoinositol and microglial activation. Datta et al. [25] reported that MRS myoinositol levels were not associated with TSPO PET in Multiple Sclerosis (MS) patients, and studies on Alzheimer’s disease have proposed that myoinositol levels might be associated with amyloid plaque load instead of microglial activation [16, 26].

Administration of the neurotoxin lipopolysaccharide (LPS) is a standard approach to activate microglia through binding to the Toll-like receptor 4, which dose-dependently induces secretion of inflammatory mediators [27]. Previously, we showed that a weak peripheral dose of LPS (1 mg/kg iv) led to a rise in myoinositol levels within four hours in an APP/PS1 model of Alzheimer’s disease, but without microgliosis or morphological evidence of microglial activation [21]. This myoinositol increase was apparent in the APP/PS1 which failed to show a microglial response to LPS, but not in wild type controls, which did show a microglial response. This raised doubts whether myoinositol really was a glial marker in AD, as previously proposed by Murray et al. (25), but questions remain regarding the robustness of the peripheral LPS challenge given the poor brain penetration of LPS [28]. Liu et al. reported CEST measurements following LPS injections in subcutaneous tumors (after a 1 mg/kg direct injection), but found no significant differences in the CEST signal one day after LPS administration [29]. No study has set out to investigate whether CEST MRI could be used to visualize changes following an LPS challenge directly in the brain.

In this pilot study we therefore aimed to explore whether CEST can detect the response to LPS-induced microglia activation in APP/PS1 transgenics and wild type controls. We expected that a challenge at a relatively low dose would be representative of the early inflammatory response present in many neuroinflammatory disorders and it would lead to differential levels of microglial activation in the AD model compared with wild-type (WT) controls. We further hypothesized that CEST MRI would allow to distinguish between these responses. To explore the origin of this CEST signal we measured a range of metabolite levels and in particular myoinositol measured by MR spectroscopy. Indeed, we found distinct responses of microglial activation through histology and CEST MRI could be used to differentiate them. However, the LPS challenge was highly variable and separation between animals with and without responses did not align with genotype.

## Methods

### Ethical statement

All procedures were approved as required under the UK Animals (Scientific Procedures) Act 1986 (Home Office project license number 40/3601). Data are reported according to the ARRIVE guidelines for *in vivo* experiments [30].

### Study design

Experimentally naïve female APPswe/PS1dE9 (APP/PS1) transgenic and wild-type C57BL6/J (WT) mice were bred in the University Biomedical Service Unit (age: 3.0 ± 0.5 months old; weight: 23.0 ± 0.6 g). They were maintained in individually vented (IVC) cages under standard husbandry conditions on a 12/12 h light cycle, with lights on at 07:00 h; the room temperature, relative humidity and air exchange were automatically controlled. Animals were group-housed with *ad libidum* access to food and water, and provided with nesting material and a play tube.

Throughout the experiment, animals were kept under anesthesia with a mixture of oxygen and isoflurane (Isocare, 3% for induction and 1–2% for maintenance). Animals were positioned in a stereotactic head frame and a small hole was made in the skull using Bregma as reference at the following coordinates: - 2 mm anterior-posterior, 1.8 mm lateral (both hemispheres), and 1 mm dorsoventral. Mice were given an intracerebral administration of lipopolysaccharide (LPS, 2 μL of 5 ng/μL) and phosphate buffered serum (PBS) contralaterally as vehicle control, before being transferred to the MR imaging system. Injections were carried out by two experimenters. MR experiments were carried out on a horizontal 9.4 T system (Agilent, Palo Alto, California) with Vnmrj 4.0 software. A 22 mm volume coil (Rapid Biomedical GmbH, Rimpar, Germany) was used for excitation and signal detection. Animals were positioned in the imaging system in a custom made frame to minimize movement. Eye gel (Lubrithal) was applied in both eyes to avoid desiccation. Body temperature and breathing rate (80–120 breaths/min) were monitored and maintained stable throughout the experiment. Brain metabolite levels were measured with MR spectroscopy, in two target volumes centered over the injection sites in the hippocampus, first on the LPS side, then, with 0.5h delay on the vehicle side where PBS was injected. Between the MRS scans, a CEST image was acquired with the imaging plane covering the injection sites. MR spectroscopy scans acquired on the vehicle (PBS) and LPS side were acquired 0.5h apart, so that the metabolite levels derived from the MR spectra would be at equal interval from the CEST scan acquisition time. Three hours after the start of the experiment mice were given an overdose of anesthesia, and brains were immediately extracted and immersed in 4% paraformaldehyde (PFA) for further processing for histology.

### Drug treatment

10 ng lipopolysaccharide (LPS, Escherichia coli serotype, Sigma0111:B4, Sigma Aldrich, in phosphate buffer saline) in 2 μL of PBS were administered through stereotactic direct injection in the hippocampus, followed contralaterally by PBS as a vehicle control, using a 5 μL Hamilton Neuros syringe (33 gauge Syringe, Sigma Aldrich).

### MR spectroscopy acquisition

For positioning of the MRS voxels, a coronal anatomical scan was acquired with a Fast Spin Echo Multi Slice (fsems) (RARE factor 8, TE = 11.8 ms, TR = 5 s, matrix size 256 × 256, field of view, FOV 15 × 20 cm, 30 slices, slice thickness 0.5 mm)[31]. Two voxels of 8 mm^3^ were centered on the hippocampus, ipsilateral and contralateral to the injury. To optimize field homogeneity, shims were first adjusted with a global field map based shim (ge3dshim, Vnmrj 4.0), followed by a local shim (FASTMAP)[32]. Shim quality was evaluated before every MRS acquisition by measuring the signal full width half maximum (FWHM) of the water peak using a LASER (localization by adiabatic selective refocusing) sequence without water suppression [33]. Shims were further adjusted if necessary. *In vivo* MR spectroscopy scans were acquired from two cubic 8 mm^3^ voxel with a LASER sequence (TR/TE = 2500/24 ms, 512 averages, 8 dummy scans, 4096 acquisition data points, spectral width 4006 Hz, acquisition time 22 min) with VAPOR (variable pulse powers and optimized relaxation delays water suppression) [34]. A reference scan without water suppression was acquired for eddy current correction. To minimize motion artifacts and frequency drift, 512 averages were acquired in pairs of two and aligned during post processing with the water peak as reference.

### MRS data analysis

Data were analyzed using LCModel for estimation of metabolite concentrations [35]. The analysis window chosen was 0.2 ppm to 4 ppm. The signal was pre-processed with eddy current correction, zero-order phasing, referencing and residual water line removal. Spectra were fitted to a linear combination of 17 metabolites in a simulated basis set containing alanine, aspartate, creatine, phosphocreatine, γ-aminobutyric acid, glucose, glutamine, glutamate, glycerophosphorylcholine, phosphorylcholine, glutathione, mI, lactate, NAA, n-acetyl aspartatyl glutamate, *scyllo*-inositol and taurine. Spectra were considered of sufficient quality if the linewidth reported by LCModel did not exceed 25 Hz. Metabolite concentrations derived from fitted spectra consistently within Cramér-Rao bounds < 10% were included in further analysis. Relative metabolite concentrations were expressed as the ratio to total creatine. Across all spectra, the average metabolite line width (full width at half maximum) was 12.9 ± 0.8 Hz, and the signal to noise ratio was 8.9 ± 0.6, as reported by LCModel.

### CEST MRI acquisition

Each CEST experiment consisted of three scans: a reference image, a CEST image array and, for B_0_ correction, a WASSR image array (water shift saturation referencing) [36]. CEST parameters were optimized based on a simulation with a three-pool model of coupled Bloch equations [37–39] using custom made software (MATLAB, The MathWorks Inc., Natick, MA), and by *in vitro* experiments. For CEST presaturation, a 1.6 s hard pulse (0.9 μT) was applied at 40 frequency offsets between ± 4 ppm at fixed intervals of 0.2 ppm. For image readout, a single-slice segmented gradient echo sequence with a Gaussian excitation pulse and centric encoding was acquired in two segments with 64 phase encoding steps each (FOV = 20 x 20 mm, slice thickness 2 mm, matrix size 128 x 128). The acquisition time for the CEST image array was 6 min. The WASSR array consisted of a 0.5 s hard prepulse (0.1 μT), followed by the segmented GE readout with 35 offsets in ±1 ppm (acquisition time 5 min). The reference image was a single segmented GE acquisition without pre-saturation. In order to improve magnetic field homogeneity, a 3% agarose gel filled the space between the top of the head and the volume coil [40]. Shimming for CEST MRI was done using an automated 3D gradient echo shim procedure (ge3dshim, Agilent). A linewidth < 30 Hz in the slice to be imaged was deemed acceptable. Manual shims were used when required.

### CEST image analysis

CEST images were analyzed with custom made software (Matlab, The Mathworks, Nattick, MA). Images were thresholded, normalized and for each voxel, z-spectra were frequency-interpolated using a spline method. WASSR frequency shift maps were derived with the maximum symmetry algorithm and used to correct CEST image arrays [36]. This resulted in a normalized z-spectrum M_sat_/M_0_ and an asymmetry spectrum MTR_asym,_ expressed in %:

$${MTR}_{asym}(\Delta \omega )=100*\;(\frac{M_{sat}(-\Delta \omega )}{M_{0}}-\;\frac{M_{sat}(\Delta \omega )}{M_{0}})$$

where M_sat_ is the MR signal intensity measured with the CEST sequence, M_0_ is the reference signal intensity, and Δω the frequency shift.

CEST MTR_asym_ values for each offset frequency were expressed as the average contrast in 1.25 x 1.25 mm regions of interest (ROI) drawn on a CEST map, one on each side covering the LPS or PBS injection sites. The size and position of the CEST ROI was chosen to match those for the quantitative analysis of the immunohistochemistry slides centered on the hippocampus. The B_0_ homogeneity between LPS and vehicle was deemed sufficient for further analysis if the frequency difference between the B_0_ measures in the LPS and vehicle ROI did not exceed 0.05 ppm.

### Immunohistochemistry

Brains were fixed in 4% paraformaldehyde (PFA), stored at 4–8 °C for a minimum of 48 hours, and then embedded in paraffin wax on a tissue embedding station (Leica TP1020). 7 μm-thick coronal sections were cut throughout the hippocampus using a microtome (Microtome Slee Cut 4060), mounted on APES coated slides and dried overnight at 40°C. Immunostaining of Iba1 (ionized calcium binding adaptor molecule 1) was carried out as previously described (26). Sections were first re-hydrated in consecutive rinses in xylene, 100% ethanol, 70% ethanol and dH_2_O and then immersed in sodium citrate buffer for 20 minutes at 95–99°C for antigen retrieval. Once the solution was cooled down to 70°C, sections were washed in PBS, incubated in 1% H_2_O_2_ solution to inhibit endogenous peroxidase activity, washed in PBS and blocked in 5% goat serum. Brain slices were then incubated with rabbit anti-Iba1 (Wako, cat. nr. 019–19741; 1:6000 in PBS-T) antibody for 1h at room temperature, washed in PBS, and incubated with biotinylated secondary antibody (Vectastain Elite ABC Kit, Rabbit IgG, Vector Labs, Burlingame, CA cat. nr. PK-6101, 1:200 in PBS-T) for 30 min. Tissue was washed, exposed to ABC-HRP (Vectastain Elite ABC Kit R.T.U, Vector Labs, cat. nr. PK-7100) and labelled with DAB peroxidase substrate (Vector Labs cat. SK-4100) according to manufacturer’s instructions. Brain slices were then counterstained using a haematoxylin & eosin protocol, dehydrated in increasing concentrations of alcohol and xylene for 2min, and mounted using Clearvue mountant (Thermo Scientific, cat. nr. 4212). Digital focused photo-scanning images were then acquired using a Hamamatsu NanoZoomer-XR 2.0-RS C10730 digital scanning system with TDI camera technology a NanoZoomer (Hamamatsu Photonics K.K. Systems, Japan) at 20x magnification and visualised using NDP.view2 (NanoZoomer Digital Photography).

### Semi-automated analysis of Iba-1 immunostaining

For extraction of morphometric features, we used our own custom made software [21, 41]. A region of interest (ROI) was drawn on digitized histology images at 20x magnification, outlining the region covered by the CEST ROIs in the hippocampus (1.25 mm x 1.25 mm). Microglial cell soma were first automatically identified by blurring with an average filter of adjacent pixels and thresholding adapted for uneven background staining. Images were inspected and corrected manually to avoid artifacts. This process provided the area occupied by glial cells and isolated microglial processes and the soma size.

### Experimental outcome measures and statistics

Primary outcome measures from CEST MRI were the MTR_asym_ values from the LPS and PBS side. Primary outcome measures from MRS were the normalized metabolite concentrations of mI in the LPS and contralateral vehicle voxel. The visual inspection of activated microglia by the pathologist was based on the number and morphology of Iba-1 stained microglial cells, comparing cells in the hippocampus across both brain hemispheres. As confirmation of the expert assessment, the difference in Iba-1 stained microglia soma size per slice was used as in our previous study [21] as a morphometric marker of microglia activation known to be sensitive to LPS [42, 43]. Data are presented as median (interquartile range) and were statistically analysed using R (version 3.4.4) [44]. Boxplots represent the median, interquartile range and outliers denoted by single points or whiskers. Outcome measures were compared between the ROIs at the site of LPS injection and the contralateral ROI (PBS vehicle control) using both a paired t-test and a nonparametric Wilcoxon rank sum test. Experiments and data analysis were performed in blind.

Primary data are available on the Imperial College Research Data Repository, under 10.14469/hpc/4127, while analysis tools are available for download on Github (https://github.com/MariaICL/Paper_analysis_scripts).

### Group sizes and exclusions

Of initially 17 mice in the pilot study, three were excluded from the analysis due to poor quality of histological staining, which did not allow assessment of microglial morphology. MRS linewidth limits were exceeded in two animals, so their MRS data was excluded from final statistics. One APP/PS1 animal showed a larger response on the vehicle side than the LPS side, and one WT animal was injected outside the hippocampus in the striatum. Both are reported separately. Table 1 summarizes the groups included in the analysis.

**Table 1:** Group sizes in analysis.

Response to LPS ^1^	All	WT	APP/PS1
Iba1-	**6**	3	3
Iba1+ ^2^	**6 (4)**	3	3 (1)

^1^ measured by immunohistochemistry. Group sizes after exclusions of animals; two animals reported separately.

^2^ in two animals, MRS but not CEST measures had to be excluded from further analysis. MRS group sizes shown in brackets.

## Results

### Microglial response to the inflammatory challenge

Histology was used to establish the response to the inflammatory challenge. All histological images were examined in blind by an experienced, board certified pathologist. A highly variable response was observed to the immune challenge, which could be readily distinguished in two categories: those animals with strong activation of microglia [41] on the side where LPS was administered (LPS Iba1+, n = 6, 3 WT, 3 APPSwe/PS1), and those who did not show a response (LPS Iba1-, n = 6, 3 WT, 3 APPSwe/PS1), judged by the morphometry of the microglial cells. One animal appeared to show higher activation in four of five slides on the PBS injection side (PBS Iba1+, S1 Table) and is excluded from statistics and reported separately. The qualitative assessment by the pathologist matched the independent quantitative assessment of microglial activation when microglial soma size was measured.

**Fig 1:**
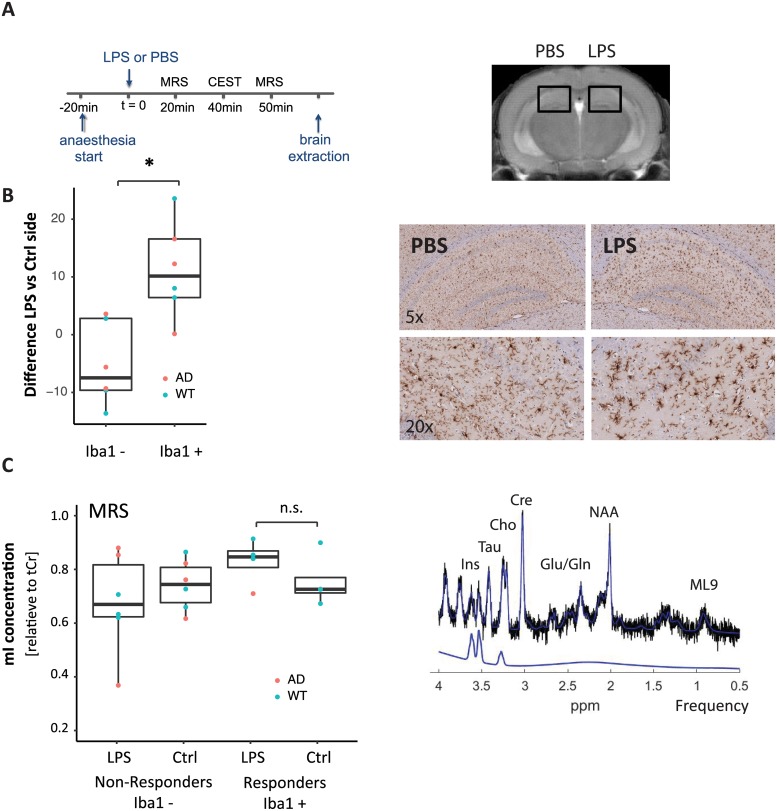
Microglial activation after LPS immune challenge. (A) Experimental protocol: Animals were anesthetized, injected with LPS and contralaterally vehicle into the hippocampus, before CEST and MR spectroscopy scans were acquired in a region covering the injection sites. At the end of the experiment, tissue was processed for histology. (B) Immunohistochemistry: Iba-1 staining revealed stronger microglial activation at the site of the injection (right) in a subset of animals. Responders to the stimulus (Iba1+, n = 6) had stronger microglial activation on the LPS side compared with an ROI on the contralateral hemisphere (p < 0.005). This difference was not seen in the other group of animals (Iba1-, n = 6). (C) MR spectroscopy: A typical spectrum (from volumes delineated in Fig. 1A) is shown on the right with a myoinositol fit curve. There were no significant differences in relative myoinositol levels for either Iba1+ (n = 4, following linewidth exclusions) or Iba1- (n = 6).

In the Iba1+ group, Iba-1 immunostaining showed a strong localized microglial response to LPS, which was higher compared with the contralateral side, where vehicle (PBS) only was administered (Fig 1, S1 Fig). For Iba1+ the difference in soma size between the LPS vs the contralateral PBS side was 10.1 μm^2^ (interquartile range: 6.8–15.5 μm^2^, p = 0.0041 paired t-test, p = 0.0087 Wilcoxon rank sum test). For Iba1-, the difference was -7.5 μm^2^ (interquartile range: - 9.6–0.7 μm^2^). No differences in classification in response to the stimulus were observed between genotypes (Fisher exact test, p >>0.05). However, we observed a difference with respect to the experimenter carrying out the intracerebral injection, and the Iba+ animals were among the first to receive the LPS challenge (S1 Table). Injection sites were not visible in T2 weighted images but could be identified in T2 maps. However, average T2 values did not differ between the site of LPS and vehicle administration (S2 Fig).

### Inflammatory response measured by CEST and MRS

We tested our hypotheses based on these categories (Iba1+, Iba1-); in particular, we hypothesized that CEST at ~0.6 ppm is sensitive to the immune challenge, and that MRS of myoinositol is a marker of this activation.

#### CEST response to LPS is higher in Iba1+ animals

**Fig 2:**
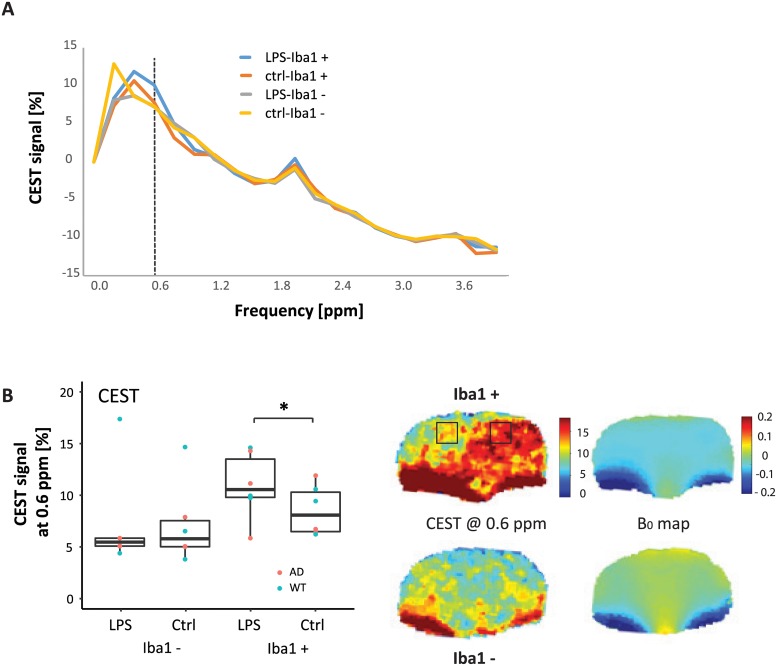
CEST MR imaging. (A) Mean CEST signal asymmetry (MTR_asym_) plots for each group (Iba1+, n = 6, and Iba1-, n = 6) from the ROI centered on the LPS injection site, and the contralateral PBS vehicle. (B) In Iba1+ animals, the CEST signal at 0.6 ppm on the LPS side (LPS Iba1+) was higher than on the contralateral vehicle side, where vehicle only was injected (PBS Iba1+, n = 6, paired t-test, p < 0.05). The CEST images at 0.6 ppm show the difference between the hemispheres, but also show artefacts in the lower part of the brain due to B_0_ inhomogeneity, visible in the B_0_ maps.

To evaluate whether Iba1+ and Iba1- animals differed in their CEST response, we acquired CEST images covering the frequency range ± 4 ppm. MTR_asym_ curves differed most in the range < 1ppm. In Iba1+ mice, the MTR_asym_ curve at 0.6 ppm was higher on the side of the LPS administration than in the contralateral vehicle control region where vehicle was injected (LPS side: 10.7% (interquartile range 10.0–13.7%); PBS side: 8.2% (interquartile range: 6.6–10.5%; n = 6, p = 0.043 paired t-test, p = 0.094, Wilcoxon rank sum test; Fig 2). Iba1- mice did not show a significant difference (LPS side: 5.6% (interquartile range 5.2–5.6%); PBS side: 5.9% (interquartile range: 5.1–7.7%; n = 6, p = 0.88 paired t-test, p = 0.84 Wilcoxon rank sum test). The CEST images in Iba1+ animals show a higher CEST signal on the LPS side, but also reveal artefacts in regions of B_0_ field inhomogeneities.

#### Myoinositol levels after LPS

To test whether myoinositol levels in the brain were changing in response to the inflammatory challenge, we acquired two MR spectroscopy scans, one centered on the LPS injection site, and one on the contralateral PBS vehicle side (Fig 1 and S1 Fig). While in Iba1+ animals, mI levels relative to tCr on the side of the LPS injection tended to be higher compared with the contralateral vehicle side, these differences were not significant (LPS side: 0.82; interquartile range: 0.78–0.84; PBS side: 0.70; interquartile range: 0.69–0.74; n = 4, p = 0.21, paired t-test, p = 0.38, Wilcoxon rank sum test, n.s.). No significant difference between hemispheres was observed in Iba1- animals, where relative mI levels on the LPS side were 0.64 (interquartile range: 0.60–0.79) and on the PBS side: 0.72 (interquartile range: 0.65–0.79; n = 6, p = 0.56, paired t-test, p = 0.69, Wilcoxon rank sum test, n.s.). In post-hoc analysis, we also did not observed significant differences between LPS and vehicle control for glutamate, glutamine, creatine, or choline.

One animal, a transgenic APPSwe/PS1, did not align with the categorization in Iba1+ vs Iba1- after LPS administration, but showed more activation on the side where the control vehicle was injected (difference in soma size between LPS vs PBS side -0.15 μm^2^). In this animal, mI levels relative to tCr were 0.63 (LPS side) vs 0.63 (PBS side), while CEST MTR_asym_ levels were 3.96% (LPS side) vs 2.98% (PBS side). In another animal, the injection was performed in the striatum instead of the hippocampus, with microglia activation present in the LPS side (difference in soma size between LPS vs PBS side 3.4 μm^2^). In this animal, mI levels relative to tCr were 0.61 (LPS side) vs 0.51 (PBS side), while CEST MTR_asym_ levels were 6.3% (LPS side) vs 4.7% (PBS side).

## Discussion

In this pilot study, we used intracerebral low dose LPS as an acute inflammation challenge in young wild type and AD mice. We designed the pilot study to explore whether CEST can index activation of microglia with this inflammatory challenge, and whether at these early stages of disease, MRS and CEST biomarkers would be sensitive enough to distinguish differential levels, if present, of activation in WT and AD animals. While an LPS challenge is an established procedure, we found the immune response at this dose to be highly variable; unilateral activation of microglia was seen by immunohistochemistry only in a subset of animals (‘Iba1+’).

To test whether CEST MRI could be a specific imaging biomarker to monitor the inflammatory response, we chose an LPS challenge as one of the more widely used experimental routes of inducing neuroinflammation [45]. A lipopolysaccharide challenge is of particular interest in Alzheimer’s disease, since LPS may impact hallmarks of disease in mouse models of AD. Intrahippocampal LPS injections have been shown to reduce β-amyloid peptide load in transgenic AD mouse model mice [46]. Also, the LPS-derived TLR-4 ligand monophosphoryl lipid A (MPL) restricts cognitive deficits in APP/PS1 transgenic mice [47]. Moreover, LPS induces a stronger inflammatory response in AD model mice than in wild type controls [48]. We chose a direct injection into the hippocampus to allow intra-subject comparisons, and due to the poor brain penetration of LPS [28]. We chose the low dose of LPS based on our earlier *in vivo* study, where we sought to elicit a differential response between WT and AD mice [21]. Specifically, we had found that peripheral, low dose (100 μg/kg) LPS led to a significant increase in myoinositol levels within four hours in the AD transgenic mice but not in WT controls. Consequently, in the current study we had expected that the direct intracerebral challenge would lead to differential levels of microglial activation in the AD model where primed microglia may be more susceptible. However, while a subset of animals showed a pronounced response to LPS readily identifiable in immunohistochemistry, this variation did not align with genotype. A possible explanation could be that at this young age, differences between genotypes are not very significant, since for example, this model only starts to show synaptic loss by 4 months, and plaques and gliosis by around 6 months [49].

Therefore, rather than separating the groups for analysis in WT and AD, instead we grouped animals based on whether a unilateral activation was observed (Iba1+ vs non Iba1-), by an expert blind to the study examining the slides from immunohistochemistry stained for the microglial marker protein Iba-1. The assessment by the pathologist served as the gold standard for separating the groups, and this did align with the difference in soma size as a quantitative measure between the two groups. Soma size was the measure we used in our previous study as a marker of microglia activation, although more accurate histological measures would allow to determine the presence of neuroinflammation. There is a linear, albeit weak relationship between the CEST signal and the quantitative histology measures, when considering the difference between the LPS injection site and the contralateral vehicle control (S3 Fig).

In those animals, where an acute intracerebral LPS challenge led to a pronounced microglial response, the CEST MRI signal at 0.6 ppm was higher in the brain hemisphere where LPS was injected, compared with the contralateral control with vehicle injection only. In our pilot study, myoinositol levels measured by MR spectroscopy tended to be higher following LPS but did not reach significance. A possible explanation is the smaller group size with MRS data, compared to histology and CEST. Two MRS datasets from the Iba1+ group had to be excluded due to the spectral quality (linewidth criteria).

A range of *in vivo* imaging techniques based on PET or MRI have been proposed to study neuroinflammation and its spatial distribution. PET imaging of the TSPO receptor is the best characterized translational technique for imaging neuroinflammation *in vivo*. MR contrast agents have been widely used to visualize local inflammation in the brain, for example with superparamagnetic iron oxide particles [50] or with perfluorocarbons [51], which avoids background signal but lacks sensitivity. More targeted approaches to image neuroinflammation have been successful with contrast agents, for example dextran coated SPIOs, functionalized with antibodies or ligands that bind to specific targets [52]. An endogenous MR imaging biomarker that is sensitive and specific enough to detect LPS induced metabolic changes with high spatial resolution would be an important step, because once validated it could be readily transferred to clinical MR systems. Microstructural changes as a consequence of local neuroinflammation have been assessed with diffusion MRI [53] or magnetization transfer imaging [54], but these approaches still lack specificity. A range of studies have assessed spectroscopic changes [9] at the expense of spatial resolution. CEST MRI has the potential to bridge this gap, targeting specific moieties while retaining spatial resolution. However, care must be taken when interpreting our results, because of challenges due to the model and the CEST contrast itself.

Hydroxyl groups have broad CEST effects in the 0–1.5 ppm region. Other visible contributions to the CEST spectrum in our data are the amine (2 ppm) and amine (3.5 ppm) peaks. The peak at 2 ppm is generally attributed to creatine [55] and proteins [56], but did not differ between groups that showed a more pronounced response to LPS (‘Iba1+’). CEST MRI in the frequency range below 1 ppm poses several technical challenges. First, CEST resonances from hydroxyl groups are strongly affected by direct water saturation since both the CEST effect and spillover or direct saturation of the free water pool increase with B_1_ power. Direct saturation thus imposes a restriction on the RF pulse power and limits the CEST saturation efficiency. Secondly, static magnetic field (B_0_) inhomogeneities shift the water resonance frequency, which results in asymmetric direct water saturation and consequently in artefacts in asymmetry analysis. The sharp peak at ~ 0.2 ppm (vehicle control–Iba1- group) is a B_0_ artifact. Accurate correction of field inhomogeneities is crucial for CEST asymmetry measurements. After extensive shimming before each MR measurement, we used the WASSR method to map the water frequency. However, there are still changes in susceptibility in regions of tissue—air interface. We therefore filled the space between the top of the head and the volume coil with agarose to reduce susceptibility artifacts and reduce variability (S4 Fig). We only quantified CEST changes if a sufficient B_0_ field homogeneity across both regions of interest in the hippocampus was achieved. Many groups studying the APT (amide proton transfer, around 3.5 ppm) signal are using Lorentzian fitting methods [57, 58], mainly because of asymmetric magnetization transfer (MT) effects around the water peak. Asymmetric MT does not have a significant effect on the CEST contrast for frequency offsets close to the water peak. Furthermore, in cases where CEST pool line shapes have merged with that of water (as in the case of hydroxyl groups), the line shape is not Lorentzian anymore and fitting will fail [59]. Hence asymmetry analysis is most suited to our analysis even though it does not allow to extract subtle details of the signal.

We cannot simply attribute the inflammatory response to the impact of LPS, because a range of factors contribute to the inflammatory response and the signals measured by MR spectroscopy and CEST MRI. The high variability we observed in microglia activity (Iba-1) could be caused by differences in the efficiency of LPS delivery between experimenters, brain injury during injection, or for example the drug viability over time (see S1 Table). To avoid bias, we therefore based our analysis and interpretation on the resulting Iba1 activity as the starting point.

An intrinsic complication for the interpretation of the CEST signal is the overlap of different molecules in the region of the myoinositol resonance. Several groups demonstrated how metabolites that contribute to the CEST signal overlap, including glutamate, glutamine, creatine, taurine and many endogenous molecules contain exchangeable–OH therefore potentially contributing to the total CEST signal (e.g. glucose, choline and myoinositol, glycerophosphocholine, and glycoproteins) [57, 60–62], an effect that we also reproduced here (S5 Fig). Therefore, direct attribution of the observed CEST effect to a specific metabolite change would require completely isolating this from other contributions, which is difficult when studying the cascade of molecular events involved in inflammation. In our study, we measured in addition to myoinositol a range of metabolites that overlap with the CEST signal < 1ppm, but did not find any significant changes after the LPS challenge. We did also exclude an effect of the drug molecule itself on the CEST signal (S5 Fig).

The injury caused by vehicle injection into the brain alone will cause a degree of microglial activation and injection sites were visible in T_2_ maps (but not in T_2_ weighted images), although signal levels in T_2_ maps did not differ between LPS and vehicle (S2 Fig). In our experiment, we controlled the effect of the injection with contralateral vehicle administration, but this does not isolate the effect of LPS.

An improvement of the current protocol may be to acquire CEST MRI and MRS separately before and after each injection (LPS and vehicle). This would allow a more careful distinction of the effects of the intervention and allow to separate the effect of LPS and injection, but require a setup where LPS and vehicle are administered in the scanner rather than the approach chosen here where procedures are separated.

It is also important to note that the area of microglial activation in histology does not precisely fit an outline of the area of higher signal in CEST MRI. This may be due to the difficulties in clearly delineating LPS enhancement or the CEST threshold chosen, but also to differences in the volume covered by the different techniques. For the quantitative assessment of our CEST MRI images and the histology, we chose a region of interest centered on the target of the injection, which was the same size in the image plane but differed in the slice thickness (2 mm for CEST, 7 μm for histology). However, the distinction into Iba1+ and Iba1- was based on the qualitative assessment of the pathologist.

We had hypothesized that a change in the CEST signal would be accompanied by an increase in the levels of myoinositol measured by MRS. In the group of animals responding strongly to LPS, myoinositol levels tended to be higher after LPS but did not reach significance. We also tested, post-hoc, other potential inflammation markers, including glutamate, choline and creatine, but these did not show significant differences. We had expected a myoinositol increase, since in contrast to our earlier study with peripheral LPS administration, we opted for the direct LPS challenge, where initial scans suggested the change in myoinositol levels. Our study does not in itself really clarify the role of myoinositol as an MRS biomarker for neuroinflammation, partially due to the limited size of our pilot study. We initially chose myoinositol due to the number of studies pointing to its potential to index neuroinflammation, but recent work from our group and others question a direct link to microglial activation [16, 17, 22, 23]. Datta et al. [25] reported that MRS determined myoinositol levels were not associated with TSPO PET in patients with Multiple Sclerosis. The authors suggested that both measures might be related to the same common process but with different time courses, or to different processes, with histopathology results suggesting a link between myoinositol and astrocyte activation.

Future experiments could benefit from another inflammatory model. Sauvage et al. locally induced astrocytic activation through lateral injection of lentiviral vectors encoding either the human CNTF gene or the β-galactosidase gene as control [14]. This is a very robust challenge which may not be representative of neuroinflammation occurring in early stages of disease, but a similar approach may be feasible that is selective for microglia. Further validation will also be necessary, for example with another imaging modality such as with TSPO PET, or the suppression of the inflammatory response with minocycline [63].

In terms of acquisition, a chemical exchange-sensitive spin lock type of experiment (higher power and shorter irradiation) would increase the sensitivity to faster exchanging spins (such as hydroxyls), while minimizing the influence of slower exchanging spins [64]. While CEST MRI benefits from higher magnetic field strength, there is potential for hydroxyl CEST in the clinic, with recent work showing that variable saturation amplitude CEST can improve sensitivity at 3T [65].

## Conclusion

In this study, we set out to monitor the response to LPS as an inflammatory stimulus with CEST. Our data suggests that CEST MRI in the hydroxyl region is sensitive to the effect of a low dose LPS inflammatory challenge in mice that show a pronounced microglial response. The immune response to LPS was highly variable and did not align with genotype. This may be due to the variability in the response to the intracerebral LPS injection, or the young age of the animals (3 months old), which may be too early to detect differences between genotypes. The sensitivity of CEST could not be explained by changes in myoinositol levels measured by MR spectroscopy, even though they tended to be higher. If a sufficiently sensitive CEST measure for neuroinflammation could be established on a clinical scanner, it could have important implications for drug development and early diagnosis in neurodegenerative disorders.

## Supporting information

S1 Table Details of experiment and resulting classification into iba1+ animals (responders) or Iba1—Non-responders, with contributing factors experimenter and genotype). In highlighted animals, there was at least a 20% change in CEST signal between LPS and PBS side.
(PDF)

S1 Fig Response to LPS. Histology was used to divide animals into Iba1+ and Iba1- to the LPS intervention. Shown here are a larger view of an example histology slide (A) with the corresponding CEST signal (B) for an Iba1+ animal with higher levels of microglial response on the LPS injection site, compared with the contralateral vehicle injection site.
(EPS)

S2 Fig T_2_ weighted images. T_2_ weighted images were acquired for all animals, while T_2_ maps were acquired for a subset of n = 6 animals. A) Representative T_2_ weighted image, with no visible effect from the injections (edema or hemorrhage). B) Both injections were visible in T_2_ maps (increased contrast in injection sites), however T_2_ values did not differ significantly between the LPS vs vehicle injection site.
(EPS)

S3 Fig Quantitative histology measures vs CEST signal. In addition to the classification into Iba1+ and Iba1-, which was based on the judgement of an experienced pathologist, we correlated a quantitative, morphometric marker of microglia activation (soma size) with the CEST response. The difference between the LPS injection side and the contralateral vehicle control (LPS side–PBS vehicle side) showed a weak relation to the CEST signal difference.
(EPS)

S4 Fig CEST signal variability in control animals. To improve field homogeneity, we applied a gel cap to the top of the skull, which improved B_0_ homogeneity across the brain: (A) After shimming but without gel cap the B0 map, CEST map and the asymmetry spectrum from an ROI on the hippocampus; (B) with the 3% agarose gel cap. The CEST spectrum showed less artifacts close to the water peak. C) CEST measurements at 0.6 ppm were acquired in four wild type mice in the absence of any intervention and averaged across ROIs in the left and right hippocampus. CEST MTRasym at 0.6 ppm were not significantly different between right and left side: in the left hippocampus ROI the CEST signal median was 7.6% (6.2%– 8.8%), while in the right hippocampus ROI the median was 6.6% (6.3% - 6.8%, n = 4, n.s.).
(EPS)

S5 Fig Contribution of different metabolites to CEST signal. While the drug (LPS) itself does not have a measurable effect in the CEST MRI acquisition, several metabolites overlap and contribute to the CEST effect around 0.6 ppm. (A) Z spectrum and asymmetry spectrum of LPS in saline solution (5ng/μL concentration, pH 7.4). (B) Z-spectrum of several brain metabolites containing -OH groups in saline solution, at physiological concentrations (creatine 6 mM, myo-inositol 6mM, glutamate 12mM, D-glucose 4mM, glutamine 4mM, GABA 3mM).
(EPS)
