# Supplementary material for: Myoinositol CEST signal in animals with increased Iba-1 levels in response to an inflammatory challenge—Preliminary findings
Source: PLoS One. 2019 Feb 21;14(2):e0212002. doi: 10.1371/journal.pone.0212002 (PMC6383890; doi:10.1371/journal.pone.0212002)
Supplement: S1 Table — In highlighted animals, there was at least a 20% change in CEST signal between LPS and PBS side. (PDF) [file pone.0212002.s001.pdf]

mouse_ID	wax_ID	genotype	Experimenter	Classification	cest_sf (PBS)	cest_sf (LP	Diff [%]
65640	3075	WT	A	0	14.90	17.65	18%
65641	1379	AD	A	1	6.52	11.33	74%
67915	3183	WT	A	1	10.76	14.83	38%
67917	3184	WT	A	1	6.34	9.90	56%
68277	3185	WT	A	1	9.59	10.12	5%
63349	1380	AD	A	1	12.10	14.51	20%
72894	3266	WT	B	3	4.73	6.26	32%
75971	3270	AD	B	1	6.84	5.96	-13%
75670	3271	AD	B	0	8.01	5.19	-35%
75669	3272	AD	B	0	5.10	5.17	1%
76547	3274	WT	B	0	6.64	5.95	-10%
76548	3275	WT	B	0	3.89	4.47	15%
75079	3276	AD	B	0	5.15	5.96	16%
75081	3277	AD	B	2	2.98	3.96	33%
63348	3076	AD	A	3			
65642	3074	WT	A	3			
75646		WT	B	3			

classification

0 = non-activated, Iba1-

1 = activated, Iba1+

2 = PBS side more activated than LPS side (excluded)

3 = excluded
